# Supplementary figures and images for: Themes, communities and influencers of online probiotics chatter: A retrospective analysis from 2009-2017
Source: PLoS One. 2021 Oct 21;16(10):e0258098. doi: 10.1371/journal.pone.0258098 (PMC8530318; doi:10.1371/journal.pone.0258098)

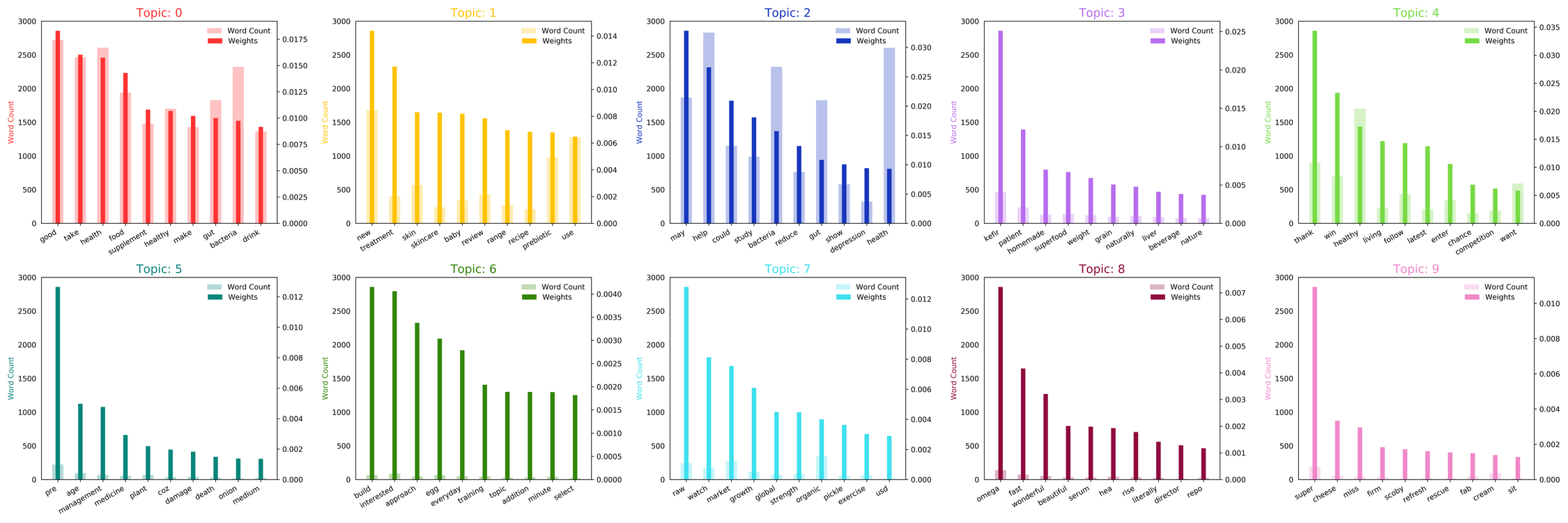

Supplement: S1 Fig — In a similar vein to PCA, higher weights equal more importance in the model. As a general rule, the frequency of the word should not significantly exceed the weight. Words that have a higher frequency relative to the weight are often less important. (TIF) [file pone.0258098.s001.tif]
