# Supplementary material for: Themes, communities and influencers of online probiotics chatter: A retrospective analysis from 2009-2017
Source: PLoS One. 2021 Oct 21;16(10):e0258098. doi: 10.1371/journal.pone.0258098 (PMC8530318; doi:10.1371/journal.pone.0258098)
Supplement: Attachment — Submitted filename: Response to Reviewers.docx [file pone.0258098.s002.docx]

Dear Editor,

On behalf of the team of authors, we wish to thank you for facilitating the peer review process and the reviewers for productive specific, constructive and detailed feedback that has strengthened our manuscript substantively. Equally, we are encouraged by their positive feedback overall and their acknowledgment of the conceptual and methodological novelty of our work.

As you can see below, we have responded to each of the reviewer’s comments in detail. Specifically, the major changes we have now made include the following: 1) the inclusion of two new metrics in the social network analyses (Average degree and Average Closeness Centrality), 2) tweaking the parameters of t-SNE model to find that the original filiform structures and clusters still hold, 3) a new paragraph outlining the implications of our study for science communication around probiotics and 4) revised the word cloud to aid in clarity, and provided detailed captions for all images to aid readers understand the figures better. In addition, we have provided detailed explanations for all of the reviewers’ questions and concerns surrounding the rigour of the methodologies used, corrected all typo errors to the best of our knowledge, and adjusted the writing where needed to aid clarity and intelligibility for the reader.

We hope that these changes have strengthened the statistical rigour of our work and enhanced its intelligibility. We are happy to incorporate any additional comments and suggestions they would be kind to offer. It has been a pleasure responding to this round of reviews and we appreciate the extension offered by journal to accommodate the situation at our end.

Thank you and we look forward to hearing from you on the revised version paper.

Best wishes,
Santosh Vijaykumar (on behalf of the authorship team)

**(Reviewers comments in black | Authors’ Responses in Red)**

**Response to Reviewer 1**

The authors have attempted to understand the interplay between content and community dynamics via online probiotics chatter, by applying topic modeling and social network analysis methods to their empirical analysis. It has been common interest to uncover evolving processes of topic-sensitive online communities across diverse research fields. The manuscript handles a significant topic, but its approaches are not sportive enough to address the topic. There are several concerns that the author needs to consider and revise accordingly.

Using Gephi, the authors conducted community detection in a giant component each year and show the basic statistics such as #nodes, #edges, and #communities in Table 1. The authors should consider diverse aspects of network properties to discuss time-evolving online communities. Only sizes of giant components and the numbers of communities are not enough to show your community dynamics. Also, ForceAtlas2 (line#215-216) is not a community detection algorithm but a graph layout algorithm.

Additionally, out- and in-degrees of a node only exhibit the shallow depth of a network (i.e. immediate neighbours), which is not enough to show influencers of a whole network. The authors are recommended to apply more comprehensive ways to figure out influencers, referring to the state-of-the-art approaches.

**Authors’ Response:** We appreciate the reviewer’s comments and thank them for the same. We have added two new metrics (Average Degree and Average Closeness Centrality) in Table 1 for all the years. These metrics help in including some new discussion points for changes through the years. Here is the excerpt for Table 1-

*“In Table 1, statistics related to the graphs/networks generated with the tweets are listed along with the communities count. Over time, we observe an increase in the number of nodes and edges in the network indicating consistently expanding communication activities around probiotics on Twitter. In terms of average degree of nodes per year, the graphs fall into two categories (until and after 2013). Until 2013, the average degree was below 2.5. In the latter category, the average degree seems to have increased with values getting close to 3. The closeness centrality metric is a way of detecting nodes that are able to spread information very efficiently through a graph. With 2009 as the exception, it is observed that this metric has consistently increased every year at an average level. The degree and closeness centrality values both indicate that probiotic Twitter users seem to be posting more tweets, with increasing proximity in the networks. We also see that the probiotics network initially starts with only two communities in 2009 but grows to nine by 2014 and settles down at six to seven by 2017.”*

We had incorrectly mentioned ForceAtlas2 as a community detection algorithm. In fact, we used the default modularity feature in Gephi to identify communities. We then used the Fruchterman and Reingold algorithm for rendering the graph in an aesthetic manner.

Here is the modified excerpt from Page 9 – *“Using Gephi’s modularity feature, nodes were classified into different modular classes in all the year graphs. This feature is based on the Louvain method [cite] for calculating modularity. We refer to each modular class as a community in this study. A total of 53 communities were identified in the nine year time period. The Fruchterman and Reingold algorithm was used in Gephi to set the layout of the graphs.36 The nodes in the graphs were sized based on their degree values.”*

This study is to analyze retrospective online probiotics chatter, but the contributions and outcomes are not quite clear. The authors kindly introduce background of probiotics, but the interlinked aspects of probiotics communities, in terms of content dynamics and community dynamics, are not clear.

We appreciate the reviewer’s feedback. We believe have substantively addressed the interlinked aspects of probiotics communities, in terms of content and dynamics from paragraphs four to eight in the introductory section; the contributions and outcomes are summarized in the Conclusion. However, if there are specific aspects of the phenomenon that the reviewer would like for us to focus on, we would be happy to consider and incorporate their suggestions.

3. Figures
Overall, figures do not help readers understand main messages. The authors need to consider different ways of showing their results with detailed captions.

**Authors’ Response**: We appreciate the reviewer’s feedback. While we have considered the reviewer’s comments and have made substantial changes to the word cloud to make it more understandable to readers. For the social network analysis, we have retained the original graphs as we believe we have chosen the most intuitive and easy-to-understand visualisations that are self-explanatory. We are happy to consider other suggestions for specific graphical formats from the reviewer that they think might be useful in better representing our findings. We have also incorporated the reviewer’s suggestion and offered more detailed captions for all figures.

**Responses to Reviewer 2**

The topic and approach is very interesting. It would be better if authors clearly described the method. It is not clear if this is a mixed method approach. Additionally, they used thematic analysis on collected tweets and it would be helpful if they described the code generation steps (how much level of agreement between coders) and showed the final codebook in a table. I am also wondering if there are sub-categories / sub-communities within each community / category. This might provide higher level of granularity.

**Authors’ Response:** We appreciate the reviewer’s comments. This paper does use two different yet complimenting analytic techniques for addressing the research questions. The topic modelling technique was used to identify the most discussed topics amongst all the tweets in the overall corpus. Social networking analysis (SNA) was required since we wanted to focus on Twitter conversations between users. Therefore, we filtered out the tweets from the corpus in which users were not tagged, for performing SNA.

Two authors were involved in an exercise for identifying names of the communities identified in the graphs. The prevalent theme of community was identified using a process involving one principal coder and one reviewer (two of the authors). First, the principal coder reviewed the contents of randomly selected tweets from each community and assigned the relevant themes.37 Next, another author reviewed and confirmed the themes assigned by the principal coder. If a community had more than one major theme. The community’s label was set to theme1 and theme2. For each of these themes, the number of constituent communities are reported. We did not use any codebook for this purpose. The community detection algorithm helps in identifying only one level of communities. Our intention was to identify a name for each community. We did not attempt to find sub-communities within the existing communities as it was not within our study scope.

In terms of the figures, we have recreated the topic models to make it more intelligible and sharper. For the SNA analysis, we have chosen the graph formats that are easiest to understand and are self-explanatory. However, if the reviewers would like to suggest any other specific formats they would prefer for us to present the findings in, we would be happy to explore those as well.

**Responses to Reviewer 3**

This is an interesting and sophisticated analysis of Twitter discussion relating to probiotics from 2009 to 2017. Extant Python tools are used to identify prominent accounts, subnetworks and topics within the wider network. Of particular interest is whether Twitter users have access to a balanced perspective (certainly an important class of question in this time of polarising echo chambers).

I hope that this can be published; beyond the interest of the findings themselves, the tools and methods will certainly be of interest to readers. In fact, with that in view, I think it would be valuable to see a bit more detail about the implementation, or perhaps to make the code used available in supplementary materials. I think it could benefit from some other revisions too. My main concern is that it's not really clearly shown how the more sophisticated analysis has given us better understanding. In the Conclusion, the actionable finding seems to be just that commercial interests are dominating online discussion of probiotics. Couldn't a much simpler analysis have told us that? Could this deeper analysis lead to specific strategic information about HOW to provide balance, e.g., by identifying the communities most in need of a scientific perspective, or identifying who public health communicators should tag to most effectively disseminate info?

**Authors’ Response**: We would like to thank the reviewer for acknowledging the novelty of our work and appreciate their critique which has prompted us to have a deeper look at the implications of our findings. We have now added these implications in the form of a new paragraph in the manner that also reflects the reviewer’s suggestions. Below is the paragraph we have added.

*“Our findings bear implications for communication strategies aimed at creating a more balanced information ecosystem about probiotic products. Specifically, apart from a few exceptions (AC 1-4) the community of probiotic scientists is clearly underrepresented on Twitter and weild minimal influence on the probiotics chatter. Twitter can be valuable to scientists in terms of disseminating their science to non-scientific audiences and engage with policymakers as well; both affordances which are of high relevance to the probiotics context^41^ given the prevailing power of advertising and labelling controversies surrounding probiotic products . Scientists can forge new networks of communication^42^ with non-academic users who have been shown in our study to weild influence in probiotics communities on Twitter . Lastly, our study demonstrates that scientists and nutrition policymakers may tag professional organisations like AN1 who, despite their seemingly limited following, may be developing growing influence in probiotics-related Twitter communities. Essentially, scientists and policymakers may imbibe the approach of commercial organisations whose efforts to grow as a Twitter authority seems to have been built on the efforts of being a hub of probiotics-related communication.”*

I have answered "Yes" when asked "Have the authors made all data underlying the findings in their manuscript fully available?" although actually all I can see is that they have stated an intention to do so, via the Open Science Foundation repository. This would need to be actioned prior to publication.

**Authors’ Response**: Yes, we can confirm that we will make all the data underlying the findings in the manuscript fully available prior to publication. For instance, we have packaged the Python scripts we used to perform the data cleaning and LDA analysis and uploaded it to [**here**](https://osf.io/756jw/?view_only=eb25517bd2394e78b792779414cdcc59)**.**

58: You refer to the favourable information as "these claims" here, but the first two sentences seem to assert them as fact. It might be more consistent if you changed "that confer" in line 54 to "that reputedly confer" or something like that.

**Authors’ Response**: We understand the reviewer’s point and thank them for the same. However, the opening sentence is the formal definition of probiotics by the FAO/WHO and hence cannot be amended. Hence, we have amended the rest of the paragraph to provide greater clarity and it now reads as follows.

*“Probiotics are defined as live microorganisms that confer a health benefit upon the host when administered in adequate amounts.^1^ Scientific evidence demonstrating its positive health effects has however been inconclusive.^2^ Because probiotics might be beneficial to individuals with specific health conditions as opposed to the general population, some regulatory agencies like the European Food Safety Authority (EFSA) have ruled against manufacturers displaying claims about the health benefits on probiotic product labels.^3,4^”*

67: I'm not sure what's meant by "expectedly." Did someone predict that this would happen?

**Authors’ Response**: What we meant by the word ‘expectedly’ was that it would be reasonable to expect that marketing of probiotic products would move online given the unprecedented rise of social media since 2008. Given the reviewer’s suggestion that this might be confused with scientific hypothesizing, we have now excluded the word ‘expectedly’ from the revised version.

92 (and similarly later): The words "let," "allow" and "enable" are synonymous, but "let" takes the bare infinitive while "allow" and "enable" take the full infinitive. Thus you can say "allow us to understand" or "let us understand," but not "let us to understand" or "allow us understand," and "enable" works the same way as "allow."

**Authors’ Response**: We thank the reviewer for this suggestion. We have now changed “allow us understand” to “allow us to understand” in two instances and “enable” in one.

105: "comprising of," while apparently fine in Singapore, is incorrect in international English; see, e.g., <https://en.wiktionary.org/wiki/comprise#cite_note-1>.

**Authors’ Response**: We have now amended “comprising of” to “comprising” in the revised version.

111: misplaced hyphen

**Authors’ Response**: Thanks for highlighting this error. It has been corrected.

111-115: These definitions should be more specific; since the in-degree and out-degree are important in this study, the reader should know exactly what they are. Does "actively communicating to other members of the network" require tagging? Do you literally mean "whether," or is this a count? It shouldn't necessarily require any more text than this to specify exactly what these quantities are.

**Authors’ Response**: We have added definitions for out-degree and in-degree in Page 11 under the Authorities and Hubs section. Here is the excerpt “In graph theory, the in-degree of a node (Twitter account in the context of this paper) is the number of incoming edges to that particular node. Similarly, the out-degree of a node is the number of outgoing edges from the particular node. In this study’s context, the edge refers to tweets where the tweets posting user is the source node and the tagged user is the target node”.

When users are being tagged with tweets, they are actively being communicated with since the tweet posting user wants the user to be involved in the conversation. We have slightly amended the sentences by adding the words “measured with” before the metric names.

169: Twitter handles are anonymised here, but later, in the Results section, we see a number of the most prominent accounts by name. I'm sure this is all legitimate (and the tweets are public, after all) but adding a few more words might clarify the matter.

**Authors’ Response**: We had initially identified individual Twitter handles in the social network analysis to aid analysis and interpretation. However, the team has now reconsidered this position in light of recent best practices surrounding the ethics of social media research and have thus anonymised the handles identified in the final analysis of the manuscript by classifying them into various categories. Our anonymisation and categorisation approach (including test of inter-rater reliability) is presented in the following paragraph.

*“Anonymisation Identification of actors facilitates the performance of and interpretation of findings from social network analysis. However, current best practices in research involving social media data (including Twitter datasets) recommend the anonymisation of users identified in the analyses due to ethical considerations.^37^ In order to reconcile this paradox, we anonymised all Twitter handles that were included in our final set of findings. We first assigned exclusive user IDs (U1, U2…) to the users originally identified in Table 4 and found N=57 exlcusive Twitter IDs. We then extracted each of their bios from their Twitter pages and performed two rounds of categorisation. In Round 1, we classified them as individuals (n=21) or organisations (n=25), accounts that ceased to exist (n=9) and those without a bio (n=2). Our review of the bios revealed that they could be further classified into discreet categories. Specifically, individuals were categorised as either academic (AC=4) or non-academic users (NAC=14); and organisations into commercial (COM=17), media (MDA=6), professional associations (AN=2) and non-profits (NPR=1). Users who could not be assigned to any of these categories were classed as others (OT=4). Following this categorisation, users were renamed sequentially by their assigned category (e.g. COM1, COM2, etc.). To ensure consistency of the categorisation scheme, two authors coded 10% of the sample (N=6) and found an initial agreement of 66.67% but achieved 100% agreement on a different sample of six tweets after clarifying the category descriptions.”*

211-217: This should be a little clearer about whether there is one giant component or a number of them (e.g., is there some threshold size, or is the giant component simply the largest connected component?), and also about what ForceAtlas2 is doing to detect communities (conceptually, if a precise description is prohibitive).

**Authors’ Response**: The giant component can be used only in a graph in Gephi. Since we loaded every year’s data separately in Gephi, the giant component was used nine times separately. The giant component feature prunes the graph to a big cluster of connected nodes where each node is connected to at least one node in the overall graph.

Regarding the community detection algorithm and graph layout method, we have made some corrections. Please find the excerpt from Page 9 –

*“Using Gephi’s modularity feature, nodes were classified into different modular classes in all the year graphs. This feature is based on the Louvain method [cite] for calculating modularity. We refer to each modular class as a community in this study. A total of 53 communities were identified in the nine-year time period. The Fruchterman and Reingold algorithm was used in Gephi to set the layout of the graphs.36 The nodes in the graphs were sized based on their degree values.”*

243: Table 2

**Authors’ Response**: Thanks for spotting the error. This has now been corrected.

245: "sence" should be "sense." More importantly, it would be good to see an example of this topic overlap.

**Authors’ Response**: I have corrected that spelling accordingly. I have also given an example of the overlap in the results section where we talk about the t-SNE as this visualises the intertopic distance in a static way.

246: "ultimaley"

**Authors’ Response**: Thanks for spotting the error. This has now been corrected.

248: This section is unclear. Its heading refers to RQ2, but the final sentence says that it "pertains to RQ1." Is there a missing "also," or is this an error? (Similar statements appear at the end of the next two sections too.) Also, what are "coders" in this context? Are they people? If so, did they view a random sample of tweets from each community, or what exactly? Did the second coder independently assign themes before comparing with the first coder's choices?

**Authors’ Response**: Thanks for highlight this error. We have removed the last sentences in both sections as they were incorrect. The term ‘coders’ refers to two of the authors. The coders did view a random sample of tweets from each community. The second coder did not independently assign the themes to the tweets, rather reviewed the assignments of the first coder.

253: This is a conditional clause, not a whole sentence.

**Authors’ Response**: Thank you for pointing out this error, it has now been fixed.

268: This sentence needs rewriting for grammar and clarity.

**Authors’ Response**: Thanks for highlighting this part. We have made the correction. The updated sentence is *“. For instance, if the accounts were closer to the y-axis in the plot, it can be inferred that such accounts post more tweets in contrast to tweets where they are tagged.”*

Figure 1: Now that I reach the figures, I note that (at least as they have reached me) the resolution is much too low. I'm sure the journal has its own process to ensure that hi-res figures are used in the final version, but as it is, I can't even read the smaller words in Fig 1. I know these word-clouds are popular, but printing them at sufficient size to make the smallest words legible will require quite a lot of space; I would consider a table.

**Authors’ Response**: This we believe may be something to do with the way the manuscript was sent for review as these figures were generated to 600dpi with a minimum font size of 24. We would like to keep the cloud as the size of the word increases as a function of its frequency. However, for the manuscript, I have reduced the total number of words to just 10 (as realistically this is what is of primary interest here) so the clouds are much more readable.

284: "the word clouds in Figure 4"? Figure 1, right?

**Authors’ Response**: Thank you for pointing this error. It does indeed pertain to Figure 1. We have now accordingly amended it.

283-285: So you're saying that the words used to define each topic tend to appear in the tweets attributed to that topic, right? That sounds inevitable, prima facie. Does this serve as a check against something that could have gone wrong? I guess what I'm saying is that it would be good to clarify this significance, if any, of the agreement between weights and frequencies within each topic.

**Authors’ Response**: With topics that are semantically related (e.g., Topic 4: promotions and Topic 7: market demand), we see in the figure that clusters of these topics emerge together. Ultimately the model has to assign each tweet with one topic, even if the words contained within it span multiple topics, which likely explains this clustering. Furthermore, the figure shows a lot of the tweets form filiform structures across the 2D plane. Our interpretation of these structures is that they may form runaway conversations (replies) and occasionally switch topic part way through. However, caution must be taken here as the dimension reduction used in t-SNE plots can lead to patterns that are exaggerated or misleading.

In a similar way to PCA in discovering latent variables, it is up to the author to name topics depending on their content. I have clarified this in this section, which I think provides the significance. 

291: "neighbouring" should be "neighbour"

**Authors’ Response**: Thank you for pointing out this error. We have now changed ‘neighbouring’ to ‘neighbour’.

297: delete a 'most'

**Authors’ Response**: Thank you for pointing out this typo; we have now deleted a ‘most’.

298: transposed L and E in 'conversely'

**Authors’ Response**: Thank you for pointing out this error which we have now fixed.

290-299: The discussion of Figure 2 is reasonable as far as it goes, but the Figure has some striking features that aren't mentioned and may disquiet the reader. In particular, the tweets seem mostly gathered into filiform structures. It's conceivably an illusion due to the dimension reduction required for the plot, but the default assumption would be that these apparent structures are meaningful. It's therefore surprising to see individual structures split up, partly allocated to one topic & partly to another. For instance, right near the top of the plot, we see five or six contiguous threads coloured partly red and partly yellow. Could a different choice of algorithm parameters in the topic allocation phase provide better agreement between the imposed partition and the finer partition that appears to be visible in this plot? Or perhaps you can offer an interpretation.

**Authors’ Response**: Many thanks for this comment, as it has prompted a more in depth look at t-SNEs and how they work. We tweaked the parameters of the model and generally found those filiform structures and clusters still existed as per the original figure. We have provided an interesting interpretation along the lines of a conversation (reply) thread, as well as provided a cautionary statement to readers regarding t-SNEs sometimes being illusions.

Your point on the overlap was like another reviewer comment on the overlap in topics mentioned in the method. These overlaps occur with semantically related topics that were picked up in the LDA model. These similar topics have common wording and the LDA ultimately assigns the strongest weighted topic for that tweet. A very similarly worded tweet however may weight the other way which provides that overlap. In an ideal world, all topics would be quite separate (like we see in Topic 0, red), but when we had models with lower numbers of topics, the coherence metrics took a real hit. Therefore, we used the compromise of decent coherence and meaningful clustering. We have added information on this in the results paragraph for figure 2 to explain.

322-330: One thing the alluvial plot shows that's not commented on here is the movement, from year to year, of Twitter accounts between different communities. It would be good to see some comment about that. While some amount of movement is probably natural (as individual account holders' interests develop), there does seem to be a surprising amount here. Does that raise red flags for the robustness of the model? Was year-to-year consistency of community membership a criterion in the community allocations, or if not, could it have been?

**Authors’ Response**: With the alluvial plot, our intention was to look for high-level trends. Hence, we did not attempt to explain the movement from year to year. The community detection algorithm was run separately for each year’s tweets. Each year’s subset of tweets has its own characteristics, and we haven’t changed anything in the data. We only tweaked the algorithm parameters in order to get the best results. The ForceAtlas2 algorithm is an industry standard and it is used by social network analysis practitioners. The year-to-year consistency of community membership was not used as a criterion in the community allocations since we did not seek to control the findings.

343: Since all the other Twitter accounts are in lowercase and preceded by an "@", "Optibac" here should be too, to avoid confusion.

**Authors’ Response**: Thanks for highlighting this. We have made the necessary correction.

383-387: If I understand the degrees correctly, then every time one account tags another, one out-degree increases by 1 and one in-degree increases by 1, i.e., the sum of the out-degrees is the sum of the in-degrees. Therefore, interpreting the proportion of accounts in which in-degree exceeds out-degree is a delicate matter, reflecting I suppose a comparison of the skews of the in-degree and out-degree distributions. I don't really understand the sentence on line 387, but I don't think it's a fair interpretation of this rather indirect observation.

**Authors’ Response**: Thanks for the comment. You are correct by saying that in any tagged conversation, the out-degree of the user who posts the tweets increases by 1 but there could be multiple users tagged in a tweet, so the in-degree of all these tagged users in that tweet would increase by 1. Hence, the sum of the out-degrees would not match with the sum of in-degrees.

With the statement “This indicates tweet posting propensity is more than being tagged in tweets”, we are making an observation from the scatter plots. It is evident that more users in the corpus since 2013 have a higher propensity of posting tweets than being tagged in others’ tweets, based on the percentage values at the top for every year. These values indicate percentage of accounts which have out-degree values more than in-degree values. Out of the nine years, five years have more accounts with higher out-degrees than in-degrees. However, the above quoted statement is not applicable for all years. Hence, we have made a slight modification – “This indicates tweet posting propensity is more than being tagged in tweets since 2013.”

401: dangling modifier

**Authors’ Response**: Thank you for pointing out the dangling modifier. We have now amended “that now” to “which” and believe this should rectify the problem.
