# Supplementary material for: Themes, communities and influencers of online probiotics chatter: A retrospective analysis from 2009-2017
Source: PLoS One. 2021 Oct 21;16(10):e0258098. doi: 10.1371/journal.pone.0258098 (PMC8530318; doi:10.1371/journal.pone.0258098)
Supplement: Attachment — Submitted filename: Response to Reviewers Rd 2.docx [file pone.0258098.s003.docx]

**Dear Editor,**

Warm greetings. At the outset, I would like to thank you for facilitating the second round of reviews. We are gratified with the overall positive responses to the major and minor changes we have made to the manuscript and are thankful to the reviewers for helping to further refine the manuscript.

In concert with the reviewers’ comments, we have now made the following changes: 1) included standard deviations for the average degree and average closeness centrality metrics, 2) discussed interlinkages between content and communities, 3) revised captions to make them more self-explanatory, 4) included descriptions of the coding process in the manuscript with additional detail, 5) provided fuller explanations of the closeness centrality measure, and 6) acknowledged the issue of in-degree vs. out-degree count in the limitations section.

We hope that these changes are satisfactory to you and the reviewers and look forward to your feedback to this revised version of our manuscript.

Best wishes,
Santosh Vijaykumar (on behalf of all authors)

**Response to Reviewers**

Reviewer # 1: The authors have improved the manuscript by incorporating the reviewers’ suggestions. There are some more recommendations that the authors need to consider as follows.

Table 1 needs to show standard deviations as well as the averages of network properties for more accurate interpretations of evolving community structures, since measured properties of all nodes unlikely follow normal distributions in the real world.

***Authors Response***: Thanks for the suggestion. As requested, we have added standard deviations for the average degree and average closeness centrality metrics in Table 1.

2) The authors are recommended to interpret the interlinked aspects of probiotic communities between dynamics of content and communities using their results from analysis.

***Authors Response***: We have now added an interpretation of the interlinked dynamics of content and communities through the following paragraph:

*The interlinkages between the dynamics of content and communities can be best understood by analysing Figure 1 (that identifies prevalent themes) in the context of Figure 3, which visualises the movement in communities across the nine years. While the top three models (Models 0, 1 and 2) suggest that Twitter chatter around probiotic products has been dominated by their characterisation as functional foods and the health benefits they offer, Figure 3 demonstrates how conversations around these health benefits have preoccupied online conversations across the study period and culminated with a surge in 2017. These findings are resonant with Burges-Watson, Moreira and Murtagh’s ^40^ qualitative observations about the “ambigious promise” of probiotic products where the benefits portrayed in popular representation such as advertising are “incommensurate” with scientific evidence. The main inference we draw from this finding, in concert with the predominance of online advertising in our dataset, is that the online information ecosystem of probiotic products might have experienced shifts in volume of chatter but have remained largely consistent in terms of content.*

The paragraphs four to eight in the intro are too general to understand the interlinked dynamics of this study.

***Authors Response***: We thank the reviewer for their feedback but have reviewed this portion and believe that the content is specific to the probiotics context. Having said so, we would be happy to consider suggestions for the inclusion of any specific literature or perspectives in this section provided by the reviewer.

3) I could not find any changes in figure captions. Figure captions need to be self-explanatory without having to read detailed content in the manuscript. Also, figure legends and resolutions are too small and low to recognize.

***Authors Response***: We have now amended the captions for all figures and hope the revised versions are satisfactory. We have formatted figure lends and resolutions in concordance with the journal’s requirements but are happy to make any specific changes we might be requested.

Reviewer #2: The authors clearly responded to most of my reviews and comments and I think this manuscript is now acceptable for publication.

***Authors Response***: Thank you very much for your positive response and the constructive reviews.

Reviewer #3: I think the article is substantially improved, and most of my concerns have been addressed. I am particularly persuaded by the new discussion on Figure 2.There is one place in particular where the authors have chosen to respond to my and the other reviewer's concerns by providing explanations in the author response, without altering the manuscript. That seems odd. If the reviewers require that explanation, then almost certainly some section of the readership would as well. I am thinking especially of the coding method. I think it's worth clarifying this more in the manuscript.

***Authors Response***: Thanks for highlighting this issue. We went over the comments that we had submitted for the previous revision, pertaining to the coding exercise. We noticed some missing points, which have now been added to the section titled “Identification of Communities (RQ2)” in Page 12. Here is the full excerpt –

*“As mentioned earlier, 53 communities were identified in the Twitter graphs built for the nine-year time period. The prevalent theme of community was identified using a process involving one principal coder and one reviewer (two of the authors). First, the principal coder reviewed the contents of randomly selected tweets from each community and assigned the relevant theme names.38 Next, another author reviewed and confirmed the themes assigned by the principal coder. It is to noted that this author did not independently assign the themes to the tweets, rather reviewed the assignments of the first coder. If a community had more than one major theme, the community’s label was set to theme1 and theme2. For each of these themes, the number of constituent communities are reported. We did not use any codebook for this exercise.”*

The addition of "closeness centrality" may be a step in the right direction, but is currently unclear. Neither a definition nor a citation for this statistic is provided, and the interpretation is confusing. It is introduced as "a way of detecting nodes that are able to spread information very efficiently," and yet it seems to be computed at either the cluster or population level rather than the node level. Thus it's mysterious how such a measure could identify which nodes spread info efficiently, and generally unclear what it's measuring.

***Authors Response***: The closeness centrality measure in Gephi is an implementation of a corresponding algorithm which was first proposed in a research paper [1]. As per Gephi’s wiki page, the development team define the metric as “the average distance from a given node to all other nodes in the network”. As per our understanding, the metric is calculated for each node in the graph. We acknowledge that the sentences in the previous manuscript pertaining to this metric, do appear inadequate. Accordingly, we have made some suitable amendments. Please find the excerpt below.

*“The average closeness centrality [1] per node, has been included in Table 1. This metric defines the importance of a node in the graph, by measuring how close the node is to other nodes in the graph (sum of geodesic distance between the particular node and all other nodes in the graph). In a graph of multiple nodes, the nodes with relatively lower closeness centrality values, are considered to be closer to the other nodes in the graph. With 2009 as the exception, it is observed that this metric has consistently increased every year at an average level. Although probiotic Twitter users seem to be posting more tweets through the years, the proximity to each other has been steadily decreasing as indicated by the rise in average closeness centrality values.”*

[1] Brandes, Ulrik. "A faster algorithm for betweenness centrality." Journal of mathematical sociology 25.2 (2001): 163-177.

Lastly, the in-degree vs out-degree count is not resolved to my satisfaction. In-degrees and out-degrees are indeed familiar from graph theory (though I think the revised explanation could be useful for part of the readership). However, in a directed graph, the total in-degree and total out-degree ARE equal. The authors seem to be comparing the number of tweets tagging an account with the number of tweets posted by an account, but in order to see those as in-degree and out-degree, we'd need to have ONE edge emerging from a tweet, and then splitting (away from any node) into MULTIPLE edges to terminate at each of the tagged accounts. This is not a standard graph-theoretic model; it's dubious that it's the best way to handle it, but certainly it's unreasonable to use this model without explaining it.

***Authors Response***: Thank you for the comment. Yes, you are right that the total in-degree of all nodes in a graph should match with the total out-degree. We would like to clarify that our comment in the earlier response letter, pertained only to singular nodes for which the in-degree and out-degree vary but at a total graph level, these metrics add up to the same value. We also acknowledge that the twitter graph representation in this paper, is not the standard graph-theoretic model. This is a limitation in this study. We have included this point a limitation in the paper. The limitations paragraph has been inserted below.

*“The generalizability of our findings is constrained by four main methodological limitations. First, by considering only tweets that contain the terms ‘probiotic’ or ‘probiotics’, our analysis could be missing other relevant tweets which do not contain these terms but might still be related to issues surrounding probiotics or probiotic supplement. Our rationale for adopting the approach we did was to use terms that would offer us both, the specificity and breadth to be able to capture the dataset of most relevance to our research questions. Second, the twitter graphs built for this study are not a representation of the standard graph-theoretic model. It is to be highlighted that we are interpreting the in-degree and out-degree values as proxy measures for tagging and posting behavior of user accounts (nodes) in the graph. Third, the analysis of tweets for identifying community theme names, could be more robust if independent coding of the tweets was conducted. However, the large number of tweets rendered this process time-consuming. Accordingly, the review and confirmation of the themes from a second coder was sought as an acceptable compromise. Finally, we analyzed data from Twitter for this study. However, users may have used other social media platforms such as Facebook and Reddit to discuss about probiotics. Thus, this study’s findings may not fully represent the overall social media discussion on probiotics.”*
